# Effects of dose titration on adherence and treatment duration of pregabalin among patients with neuropathic pain: A MarketScan database study

**DOI:** 10.1371/journal.pone.0242467

**Published:** 2021-01-20

**Authors:** Yu-Chen Yeh, Joseph C. Cappelleri, Xiaocong L. Marston, Ahmed Shelbaya

**Affiliations:** 1 Pharmerit International, Newton, MA, United States of America; 2 Department of Global Biometrics and Data Management, Pfizer Inc, New York, NY, United States of America; 3 Pharmerit International, Shanghai, China; 4 Department of Health Economics and Outcomes Research, Pfizer Inc, New York, NY, United States of America; 5 Mailman School of Public Health, Columbia University, New York, NY, United States of America; Jouf University, Kingdom of Saudi Arabia, SAUDI ARABIA

## Abstract

**Objective:**

To examine pregabalin dose titration and its impact on treatment adherence and duration in patients with neuropathic pain (NeP).

**Methods:**

MarketScan database (2009–2014) was used to extract a cohort of incident adult pregabalin users with NeP who had at least 12 months of follow-up data. Any dose augmentation within 45 days following the first pregabalin claim was defined as dose titration. Adherence (measured by medication possession ratio/MPR) and persistence (measured as the duration of continuous treatment) were compared between the cohorts with and without dose titration. Logistic regressions and Cox proportional hazards models were used to identify the factors associated with adherence (MPR ≥ 0.8) and predictors of time to discontinuation.

**Results:**

Among the 5,186 patients in the analysis, only 18% of patients had dose titration. Patients who had dose titration were approximately 2.6 times as likely to be adherent (MPR ≥ 0.8) (odds ratio = 2.59, *P* < 0.001) than those who did not have dose titration. Kaplan-Meier analysis shows that the time to discontinuation or switch was significantly longer among patients who had dose titration (4.99 vs. 4.04 months, *P* = 0.009).

**Conclusions:**

Dose titration was associated with improved treatment adherence and persistence among NeP patients receiving pregabalin. The findings will provide valuable evidence to increase physician awareness of dose recommendations in the prescribing information and to educate patients on the importance of titration and adherence.

## Introduction

### Neuropathic pain and treatment

Neuropathic pain (NeP), defined as “pain caused by a lesion or disease of the somatosensory nerve system”, is a syndrome derived from a range of etiologies such as diabetes mellitus, spinal cord injury, shingles, multiple sclerosis, tumor compression, stroke, and human immunodeficiency virus (HIV) infection[1, 2]. NeP negatively impacts patients’ quality of life (QoL) and limits their daily activities [3–5]. The estimated prevalence of NeP varies from 6.9% to 10% in the general population [6]. In terms of the economic burden of NeP, indirect costs due to absenteeism and decreased productivity were the primary cost driver, totaling $100 billion each year in the US [7]. Currently, there is no curative treatment for NeP and the goal of treatment is to achieve symptom control [8]. Based on the guidelines, tricyclic antidepressants (TCAs) (e.g., amitriptyline and nortriptyline), calcium channel alpha-2-delta ligands (e.g., gabapentin and pregabalin), and serotonin-norepinephrine reuptake inhibitors (SNRIs) (e.g., duloxetine and venlafaxine) are recommended as first-line options for NeP treatment, whereas tramadol and other opioids are considered as second-line agents [8–14].

Pregabalin (LYRICA, Pfizer), a ligand of the alpha-2-delta (α-2-δ) subunits of voltage-gated calcium channels [15], was approved by the US Food and Drug Administration for the management of NeP associated with diabetic peripheral neuropathy (DPN), post-herpetic neuralgia (PHN), fibromyalgia, spinal cord injury and adjunctive therapy for the treatment of partial onset seizures in patients four years of age or older [16]. Data from randomized controlled trials showed that pregabalin had early and sustained effect on pain relief for NeP, as well as a beneficial effect on related sleep interference [17, 18]. Pregabalin has also been proved to be well tolerated with a low discontinuation rate when titrated over one week to fixed dosages [18].

### Treatment adherence and dose titration

Whilst efficacy of medicines in clinical trials is generally well demonstrated, the effectiveness of a pharmacological treatment in clinical practice is strongly influenced by patient adherence. Indeed, nonadherence remains a challenging problem in NeP management. One Swedish study reported that more than half of the patients discontinued their NeP treatment after the first three months while 60–70% discontinued after six months [19]. Gharibian et al. explored the adherence and persistence among patients in the US who received antidepressants and anticonvulsants for NeP and noted that less than 50% of the patients were adherent or persistent during the first-year treatment [20]. Non-adherence was associated with poor disease control and increased healthcare resource utilization and costs [21–25].

Adherence to medication refers to the extent to which patient behavior matches health advice [26]. There are numerous factors that can negatively affect patient adherence, including concerns about side effects, fears of addiction, lack of sufficient patient education, and poor efficacy or tolerability [27, 28]. The aim of pregabalin dose titration is to achieve the optimal balance between efficacy and tolerability. The prescribing information suggests that pregabalin be started at 150 mg/day [29]. The dose may be increased to 300 mg/day within one week. Further increase in dose may be considered depending on the indication [29].

Studies have shown that dose titration was associated with improved pain relief. A pooled analysis of seven placebo-controlled clinical trials of pregabalin in DPN showed that increased doses were associated with greater pain relief [30]. In a recent simulation study using integrated data from nine clinical trials and an open-label observational study, researchers found that upward dose titration to 300 mg/day was associated with greater proportions of pain relief regardless of baseline pain severity in patients with DPN [31]. Serpell et al. conducted an in-depth analysis of six flexible-dose clinical trials of pregabalin in patients with NeP. The study found that many patients who do not respond to lower doses of pregabalin will respond with notable improvements in pain outcomes when the dose is escalated [32].

In addition to improved clinical outcomes, dose titration was also associated with improved medication adherence and persistence in patients with various conditions [33, 34]. However, existing research is scarce on the relationship between pregabalin dose titration and adherence and persistence among patients with NeP. The aim of this study was to improve the knowledge on real-world use of pregabalin and the effect of dose titration on treatment adherence and persistence utilizing a large commercial claims database.

## Materials and methods

This was a retrospective cohort study of NeP patients initiating pregabalin treatment during the study period.

### Materials

Data from the Truven MarketScan Commercial and Medicare Supplement Database from 2009 through 2014 were used to extract a cohort of incident adult pregabalin users with NeP (DPN, PHN, and spinal cord injury, Table 1). The MarketScan database contains claims of approximately 100 employers and 12 US health plans representing more than 30 million covered lives including employees and adult dependents. The Commercial database includes health plan data for individuals younger than 65 years of age, whereas the Medicare Supplemental database includes predominantly fee-for-service plan data for Medicare-eligible retirees aged 65 and older with employer-sponsored Medicare Supplemental plans.

**Table 1:** Demographic and clinical characteristics.

Variable	Total *N = 5*,*186*	With pregabalin dose titration *N = 933*	Without pregabalin dose titration *N = 4*,*253*	*P* value
Age				
Mean (SD)	53 (8.6)	52 (9.1)	53 (8.5)	0.010
Median (Q1 to Q3)	55 (49, 59)	54 (48, 59)	55 (49, 59)	
Range	18, 64	18, 64	18, 64	
Age group, n (%)				
18–44 years	729 (14.1%)	159 (17.0%)	570 (13.4%)	0.004
45–64 years	4,457 (85.9%)	774 (83.0%)	3,683 (86.6%)	
Gender, n (%)				
Male	2,720 (52.5%)	478 (51.2%)	2,242 (52.7%)	0.411
Female	2,466 (47.6%)	455 (48.8%)	2,011 (47.3%)	
Region, n (%)				
North Central	1,211 (23.4%)	224 (24.0%)	987 (23.2%)	0.052
Northeast	576 (11.1%)	124 (13.3%)	452 (10.6%)	
South	2,539 (49.0%)	437 (46.8%)	2,102 (49.4%)	
West	740 (14.3%)	134 (14.4%)	606 (14.3%)	
Unknown	120 (2.3%)	14 (1.5%)	106 (2.5%)	
Plan type, n (%)				
PPO	3,290 (63.4%)	589 (63.1%)	2,701 (63.5%)	0.539
HMO	583 (11.2%)	96 (10.3%)	487 (11.5%)	
POS	444 (8.6%)	91 (9.8%)	353 (8.3%)	
Comprehensive	294 (5.7%)	56 (6.0%)	238 (5.6%)	
Other or missing	575 (11.1%)	101 (10.8%)	474 (11.2%)	
Charlson Comorbidity, n (%)				
Myocardial infarction	82 (1.6%)	15 (1.6%)	67 (1.6%)	0.943
Congestive heart failure	299 (5.8%)	48 (5.1%)	251 (5.9%)	0.369
Peripheral vascular disease	429 (8.3%)	76 (8.1%)	353 (8.3%)	0.877
Cerebrovascular disease	428 (8.3%)	66 (7.1%)	362 (8.5%)	0.148
Dementia	11 (0.2%)	1 (0.1%)	10 (0.2%)	0.442
Chronic pulmonary disease	797 (15.4%)	128 (13.7%)	669 (15.7%)	0.123
Rheumatologic disease	154 (3%)	31 (3.3%)	123 (2.9%)	0.483
Peptic ulcer	58 (1.1%)	8 (0.9%)	50 (1.2%)	0.403
Diabetes without complications	3,598 (69.4%)	561 (60.1%)	3,037 (71.4%)	< .001
Diabetes with complications	2,910 (56.1%)	416 (44.6%)	2,494 (58.6%)	< .001
Paralysis	203 (3.9%)	35 (3.8%)	168 (4%)	0.777
Chronic renal failure	423 (8.2%)	61 (6.5%)	362 (8.5%)	0.046
Any malignancy, leukemia, lymphoma	364 (7%)	78 (8.4%)	286 (6.7%)	0.077
Moderate-severe liver disease	36 (0.7%)	4 (0.4%)	32 (0.8%)	0.281
Metastatic solid tumor	63 (1.2%)	15 (1.6%)	48 (1.1%)	0.226
AIDS	25 (0.5%)	1 (0.1%)	24 (0.6%)	0.068
Charlson comorbidity index (CCI)				
Mean (SD)	2 (1.8)	2 (1.8)	2 (1.8)	< .001
Median (Q1 to Q3)	2 (1, 3)	2 (1, 3)	2 (1, 3)	
Range	0, 17	0, 12	0, 17	
CCI category, n (%)				
0	814 (15.7%)	214 (22.9%)	600 (14.1%)	< .001
1	815 (15.7%)	160 (17.1%)	655 (15.4%)	
2	1,900 (36.6%)	297 (31.8%)	1,603 (37.7%)	
≥ 2	1,657 (32.0%)	262 (28.1%)	1,395 (32.8%)	
Pre-index drug use, n (%)				
Gabapentin	2,161 (41.7%)	430 (46.1%)	1,731 (40.7%)	0.003
Opioids excluding tramadol	1,334 (25.7%)	289 (31.0%)	1,045 (24.6%)	< .001
Tramadol	955 (18.4%)	185 (19.8%)	770 (18.1%)	0.219
SNRI	776 (15.0%)	161 (17.3%)	615 (14.5%)	0.030
TCA	562 (10.8%)	122 (13.1%)	440 (10.4%)	0.015
Lidocaine	468 (9.0%)	95 (10.2%)	373 (8.8%)	0.173
BTX	2 (0.0%)	0	2 (0.1%)	0.508
Concomitant drug use, n (%)				
Gabapentin	312 (6.0%)	56 (6.0%)	256 (6.0%)	0.984
Opioids excluding tramadol	452 (8.7%)	91 (9.8%)	361 (8.5%)	0.215
Tramadol	316 (6.1%)	80 (8.6%)	236 (5.6%)	< .001
SNRI	617 (11.9%)	142 (15.2%)	475 (11.2%)	< .001
TCA	307 (5.9%)	63 (6.8%)	244 (5.7%)	0.234
Lidocaine	127 (2.5%)	33 (3.5%)	94 (2.2%)	0.018
BTX	1 (0.0%)	0	1 (0.0%)	0.640
Pre-index HCRU				
Patients with any hospital admission, n (%)	1,537 (29.6%)	304 (32.6%)	1,233 (29.0%)	0.030
Number of admissions ^a^				
Mean (SD)	1.9 (1.5)	1.9 (1.5)	1.9 (1.5)	0.891
Median (Q1 to Q3)	1.0 (1.0, 2.0)	1.0 (1.0, 2.0)	1.0 (1.0, 2.0)	
Range	1.0, 18.0	1.0, 10.0	1.0, 18.0	
Length of stay ^a^				
Mean (SD)	14.7 (24.0)	14.3 (19.7)	14.8 (24.9)	0.271
Median (Q1 to Q3)	6.0 (3.0, 16.0)	6.0 (3.0, 17.5)	6.0 (3.0, 16.0)	
Range	1.0, 256.2	1.0, 119.1	1.0, 256.2	
Patients with any ER visit, n (%)	61 (1.2%)	15 (1.6%)	46 (1.1%)	0.177
Number of ER visits ^a^				
Mean (SD)	1.1 (0.4)	1.2 (0.6)	1.1 (0.3)	0.739
Median (Q1 to Q3)	1.0 (1.0, 1.0)	1.0 (1.0, 1.0)	1.0 (1.0, 1.0)	
Range	1.0, 3.0	1.0, 3.0	1.0, 2.0	
Patients with any outpatient visit, n (%)	5,119 (98.7%)	914 (98.0%)	4,205 (98.9%)	0.026
Number of outpatient visits ^a^				
Mean (SD)	16.0 (13.5)	16.0 (13.7)	16.0 (13.4)	0.682
Median (Q1 to Q3)	12.0 (7.0, 21.0)	12.0 (7.0, 21.0)	12.0 (7.0, 21.0)	
Range	1.0, 155.1	1.0, 118.1	1.0, 155.1	

NOTE:

^a^ Annualized statistics among those who had any resource use.

***Abbreviation*:** SD, standard deviation; IQR, inter-quartile range; PPO, preferred provider organization; HMO, health maintenance organization; POS, point-of-service; AIDS, adult immune-deficiency syndrome; CCI, Charlson comorbidity index; SNRI, serotonin and norepinephrine reuptake inhibitors; TCA, tricyclic antidepressants; BTX, botulinum toxin; HCRU, health care resource use.

### Study sample

**Fig 1:**
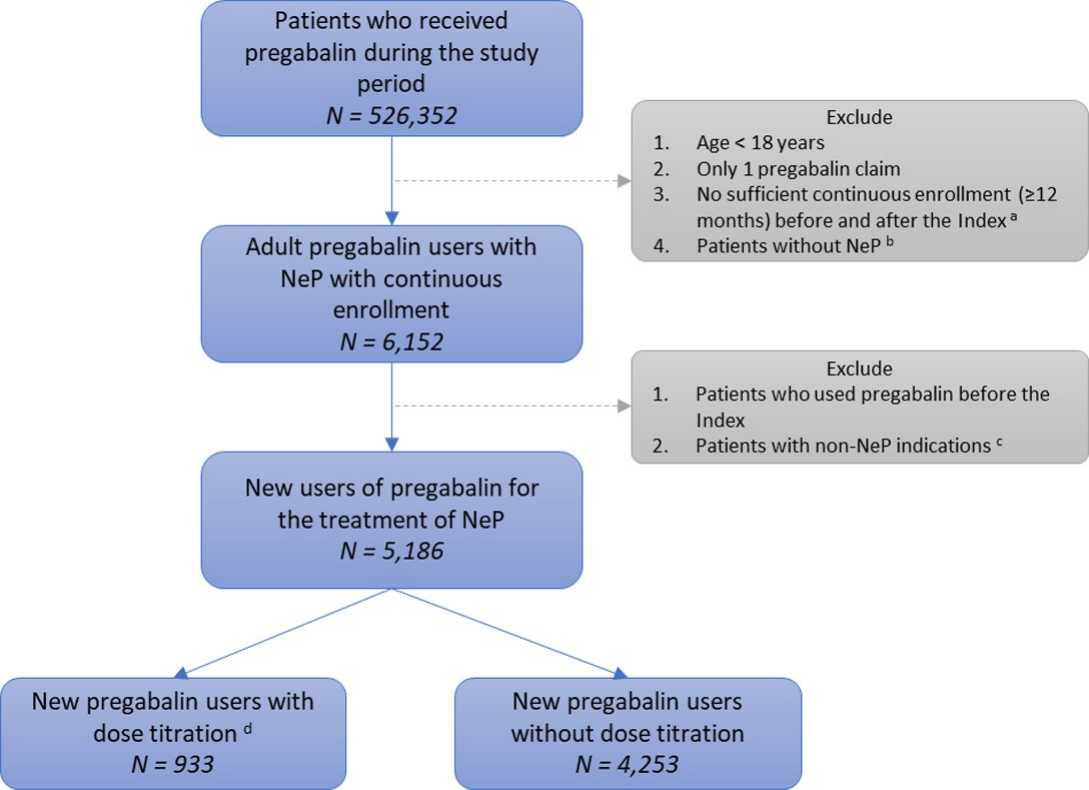
Flow chart of study sample. a. Index: first pregabalin claim. b. Neuropathic pain (NeP): diabetic peripheral neuropathy, spinal cord injury, or post-herpetic neuralgia within 12 months prior to or 1 month following the index. c. Non-NeP indications: epilepsy, fibromyalgia, amyotrophic lateral sclerosis, or multiple sclerosis. d. New pregabalin users: no pregabalin use within 12 months prior to the index.

We identified adult patients (≥18 years old) with at least 12 months of continuous enrollment prior to their first pregabalin claim, which was deemed as the index. The 12-month lookback period allowed for baseline data collection while also providing a “washout period” for identifying new pregabalin users. Eligible subjects were also required to have continuous enrollment for at least 12 months after the index pregabalin claim to observe treatment patterns. Detailed inclusion and exclusion criteria were as follows:

Inclusion criteria (Fig 1):

- At least two pregabalin claims during the study period are required within the study period to calculate treatment adherence, **AND**
- Patients with sufficient continuous enrollment (at least 12 months before and after the index pregabalin claim), **AND**
- At least one ICD-9-CM code indicating NeP (Table 2) within 12 months prior to the index pregabalin prescription or within 30 days of index pregabalin prescription, **AND**
- Age ≥18 years at index pregabalin claim.

**Table 2:** Inclusion and exclusion criteria with coding algorithms.

Criteria		ICD-9-CM	Data Period
**Inclusion**			
Neuropathic Pain			
Diabetic Peripheral Neuropathy	Diabetes with neurological manifestations	250.6	12 months before index to 30 days after index
	Polyneuropathy in diabetes	357.2	
Spinal Cord Injury (SCI)	Quadriplegia and quadriparesis	344.0	
	Paraplegia	344.1	
	Cauda equina syndrome	344.6	
	Fracture of vertebral column with SCI	806	
	SCI without evidence of spinal bone injury	952	
Post-Herpetic Neuralgia	Herpes zoster with other nervous system complications	053.1	
**Exclusion**			
Epilepsy	Epilepsy and recurrent seizures	345	Any time point
	Other convulsions	780.39	
Fibromyalgia		729.1	
Amyotrophic Lateral Sclerosis		335.20	
Multiple Sclerosis		340	

Exclusion criteria:

- Patients who used pregabalin within 12 months prior to the index claim, **OR**
- Patients who had any diagnosis related to (1) epilepsy, (2) fibromyalgia, (3) amyotrophic lateral sclerosis, or (4) multiple sclerosis (Table 2).

### Study variables

#### Dose titration

Pregabalin dose titration was a dichotomous variable, defined as any dose augmentation within 45 days following the index claim.

#### Adherence

The literature reports on a variety of adherence calculation methods based on tablet counts, patient diaries, electronic monitoring by medication containers, and use of adjudicated prescription claims from administrative databases [35]. Of methods not based on patient recall (e.g. diaries), claims-based approaches are clearly the most feasible and least costly to perform on a continuing basis. The current study assessed pregabalin adherence during continuous treatment (i.e. no gap greater than 45 days) using the medication possession ratio (MPR), a ratio of the number of doses dispensed relative to the dispensing period (See formula below). A threshold of 0.8 was used to define adherence.

$$MPR=\frac{Sum\;of\;days'\;supply\;of\;all\;pregabalin\;claims}{Total\;number\;o\;f\;days\;from\;the\;index\;date\;to\;the\;runout\;date\;of\;the\;last\;pregabalin\;claim}$$

#### Treatment switch

We measured treatment switch as non-pregabalin NeP medications (i.e. gabapentin, opioids, SNRI, TCA, lidocaine, and BTX) filled within 45 days following the runout date of a pregabalin claim (i.e., index date + days’ supply).

#### Persistence

The International Society for Pharmacoeconomics and Outcomes Research (ISPOR) Medication Compliance and Persistence Work Group defined persistence as the number of days taking medication without exceeding permissible gap [35]. In the current study, we measured pregabalin persistence using the time to discontinuation, defined as no pregabalin filled within 45 days following the runout date of a prior pregabalin claim.

#### Covariates

Covariates included patient demographics (age, sex, geographic region), type of health plan, pre-existing conditions (Charlson comorbidity index), pre-index healthcare resource utilization (hospital admission, inpatient days, outpatient visit, emergency room visit), pre-index medication use (gabapentin, opioids, SNRI, TCA, lidocaine, botulinum toxin), and concomitant use of the above medications following the index claim.

### Statistical analysis

#### Descriptive analysis

Descriptive analysis was conducted to compare the baseline demographic and clinical characteristics between patients with and without dose titration. For categorical variables (e.g., sex), Pearson chi-square tests were performed to assess statically significant difference; for sub-categories with sample sizes less than 5, Fisher’s exact test was used. For continuous variables, variances were tested using two-groups t-tests or analysis of variance (ANOVA) [36]. Kaplan-Meier survival curves were used to compare the time to pregabalin discontinuation between patients with and without dose titration [36].

#### Multivariable analysis

Multivariable logistic regressions were performed to identify factors associated with adherence (MRP ≥ 0.8). Cox proportional hazards models were conducted to identify predictors of time to discontinuation [36].

All analyses were conducted using SAS 9.4 (Cary, NC: SAS Institute Inc.). All variance analyses were 2-sided, and statistical significance was defined at α = 0.05 level.

## Results

### Study sample

Fig 1 shows the construction of the study sample. A total of 5,186 naïve pregabalin users were included in the study. There were 933 (18.0%) pregabalin users who had dose titration, while the remaining 4,253 (82.0%) pregabalin users did not have any dose augmentation within 45 days following the index claim.

### Baseline characteristics

Baseline characteristics were compared by titration status (Table 1). Compared with patients without dose titration, a lower proportion of patients with dose titration had diabetes (with or without complications) or chronic renal failure. Patients who had dose titration tended to have a lower Charlson comorbidity index, with 59.9% of patients having at least two pre-existing conditions, compared with more than 70% among patients who did not have dose titration (*P* < 0.001). Pre-index use of gabapentin, opioids (excluding tramadol), serotonin and norepinephrine reuptake inhibitors (SNRI), and tricyclic antidepressants (TCA) were more frequent among patients with dose titration versus those without dose titration. Concomitant use of tramadol, SNRI, and lidocaine was also more prevalent among patients who had dose titration. A larger proportion of patients with titration had pre-index hospitalization (32.6% vs 29.0%, *P* = 0.03).

### Patterns of dose titration

Among patients with dose titration, the average initial dose was 152 mg/day and the ending dose was 290 mg/day (Table 3), compared with 185 mg/day (*P* < 0.001) and 180 mg/day (*P* < 0.001) among patients without dose titration. Overall, approximately 1 in 4 pregabalin users (26.9%) reached 300 mg/day. The proportion of patients receiving at least 300 mg/day of pregabalin was significantly higher among those who had dose titration than those who did not (59.5% vs 19.7%, *P* < 0.001).

**Table 3:** Patterns of dose titration.

Variable	Overall *N* = 5,186	With pregabalin dose titration *N = 933*	Without pregabalin dose titration *N = 4*,*253*	P value
Initial dose (mg/day)				
Mean (SD)	179.1 (107.6)	152.4 (71.9)	185.0 (113.1)	< .001
Median (IQR)	150.0 (131.6, 225.0)	150.0 (100.0, 150.0)	150.0 (150.0, 225.0)	
Range	25.0, 1000.0	25.0, 600.0	25.0, 1000.0	
Ending dose (mg/day)				
Mean (SD)	200.1 (121.5)	290.4 (133.8)	180.3 (109.1)	< .001
Median (IQR)	150.0 (150.0, 300.0)	300.0 (200.0, 300.0)	150.0 (120.0, 225.0)	
Range	25.0, 1000.0	45.0, 1000.0	25.0, 1000.0	
Reaching 300 mg/day, n (%)	1,394 (26.9%)	555 (59.5%)	839 (19.7%)	< .001

***Abbreviation*:** SD, standard deviation; IQR, inter-quartile range.

### Association between titration and adherence

Patients who had pregabalin dose titration were approximately 2.6 times more likely to be adherent (MPR ≥ 0.8) than those who did not have dose titration [odds ratio (OR) = 2.59, *P* < 0.001] (Table 4). Other covariates positively associated with being adherent include increased age, male gender, region as north central, pre-index gabapentin use, pre-index TCA use, concomitant lidocaine use, and pre-index inpatient days. Females (OR = 0.76, *P* < 0.001) and patients with higher CCI (OR = 0.94, *P* < 0.001) were less likely to be adherent.

**Table 4:** Logistic regression for factors associated with adherence (MPR≥0.8).

Variable	Odds Ratio (95% CI)		P-value
Age	1.02	1.01, 1.02	<0.001
Region			
South	Ref		
North Central	1.24	1.08, 1.43	0.003
Northeast	1.15	0.96, 1.39	0.135
West	2.06	1.40, 3.02	<0.001
Unknown	1.16	0.98, 1.38	0.091
Insurance type			
PPO	Ref		
POS	0.89	0.72, 1.08	0.238
HMO	0.93	0.78, 1.12	0.455
Comprehensive	0.97	0.75, 1.25	0.805
Others or missing	1.07	0.89, 1.29	0.455
Gender			
Female vs. Male	0.76	0.67, 0.85	<0.001
CCI	0.94	0.91, 0.97	<0.001
**Pregabalin dose titration**	**2.59**	**2.22, 3.02**	**<0.001**
Starting Pregabalin dose >150 mg	1.06	0.94, 1.20	0.341
Pre-index opioids use (excluding tramadol)	1.01	0.88, 1.16	0.893
Pre-index gabapentin use	1.20	1.07, 1.36	0.002
Pre-index lidocaine use	0.90	0.74, 1.10	0.319
Pre-index TCA use	1.25	1.04, 1.51	0.016
Pre-index SNRI use	1.09	0.92, 1.30	0.308
Pre-index tramadol use	0.96	0.83, 1.12	0.615
Concomitant SNRI use	0.91	0.76, 1.10	0.347
Concomitant gabapentin use	0.88	0.69, 1.11	0.276
Concomitant opioids use (excluding tramadol)	1.15	0.94, 1.42	0.181
Concomitant tramadol use	1.05	0.83, 1.34	0.687
Concomitant lidocaine use	1.50	1.03, 2.19	0.035
Pre-index number of hospital admissions	1.02	0.95, 1.09	0.634
Pre-index number of outpatient visits	1.00	1.00, 1.01	0.157
Pre-index number of ER visits	0.84	0.53, 1.32	0.450
Pre-index inpatient days	1.01	1.00, 1.01	0.004

NOTE:

***Abbreviation*:** MPR, medication possession ratio; PPO, preferred provider organization; HMO, health maintenance organization; POS, point-of-service; CCI, Charlson comorbidity index; SNRI, serotonin and norepinephrine reuptake inhibitors; TCA, tricyclic antidepressants; ER, emergency room.

### Association between titration and persistence (no switch or discontinuation)

**Fig 2:**
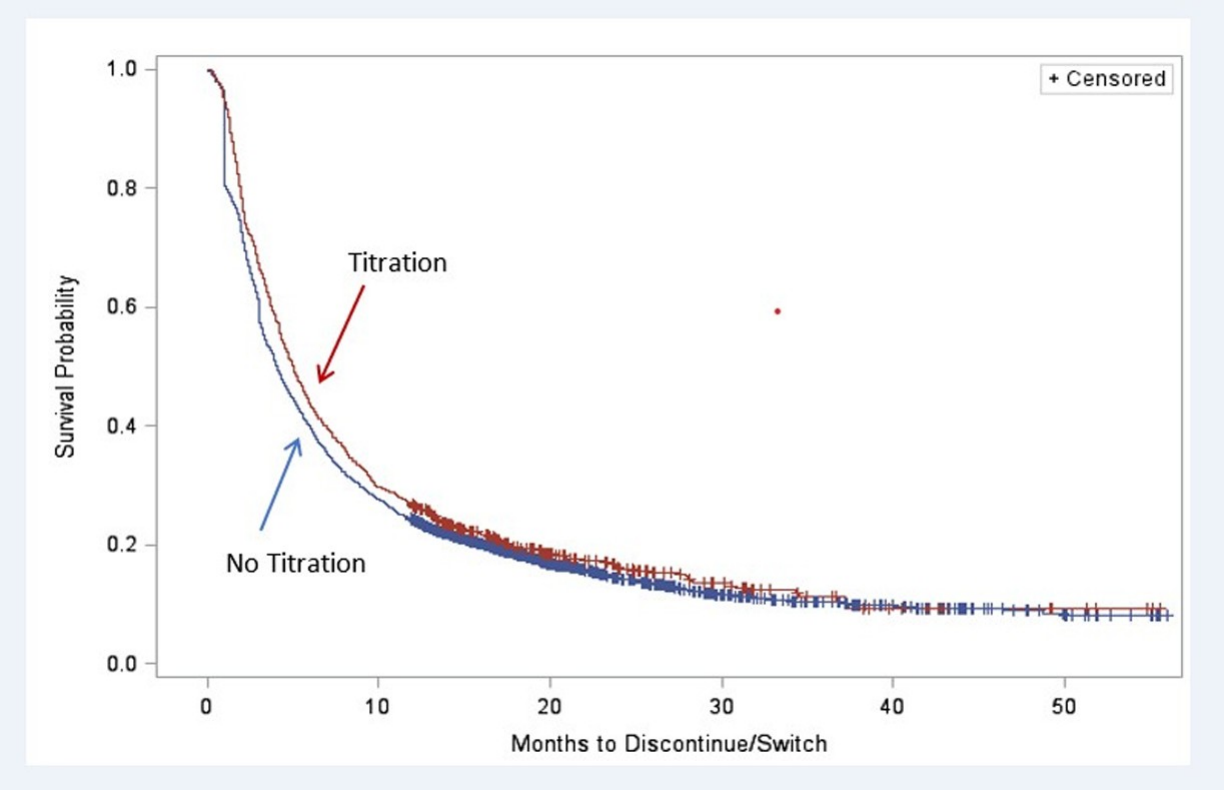
Kaplan-Meier analysis of months to pregabalin discontinuation or switch.

The median time to discontinuation or switch was 5.0 [95% confidence interval (CI): 2.1–13.1] months for those with dose titration, compared with 4.0 (95% CI: 1.9–11.4) months for those without dose titration. Kaplan-Meier analysis (Fig 2) shows that the time to discontinuation or switch was longer among pregabalin users who had dose titration (*P* = 0.009). The multivariable adjusted Cox proportional hazards model shows that pregabalin users who had dose titration had greater persistence (i.e., less likely to switch therapy or discontinue pregabalin) than those who did not have dose titration (hazard ratio = 0.91, *P* = 0.016) (Table 5). Other covariates that were independently associated with greater persistence include: age, male gender, West region, CCI score, pre-index gabapentin use, concomitant SNRI use, concomitant opioid use, and pre-index inpatient days. Pre-index use of tramadol was associated with lower persistence.

**Table 5:** Cox proportional hazards model for factors associated with treatment discontinuation or switch.

Variable	Hazards Ratio (95% CI)			P value
Age	0.99	0.99	1.00	< .001
Region				
South				
North Central	0.93	0.86	1.00	0.055
Northeast	0.96	0.87	1.06	0.424
West	0.52	0.42	0.66	< .001
Unknown	0.85	0.77	0.93	0.001
Insurance type				
PPO				
POS	1.01	0.90	1.12	0.915
HMO	0.95	0.87	1.05	0.352
Comprehensive	0.94	0.82	1.07	0.330
Others or missing	0.90	0.81	1.00	0.042
Gender				
Female vs. Male	1.20	1.13	1.28	< .001
CCI	0.97	0.95	0.99	0.004
**Pregabalin dose titration**	**0.91**	**0.84**	**0.98**	**0.016**
Starting Pregabalin dose >150 mg	0.94	0.88	1.00	0.052
Pre-index opioids use (excluding tramadol)	1.06	0.98	1.14	0.133
Pre-index gabapentin use	0.85	0.80	0.91	< .001
Pre-index lidocaine use	1.11	1.00	1.23	0.061
Pre-index TCA use	0.92	0.83	1.01	0.091
Pre-index SNRI use	0.97	0.89	1.07	0.578
Pre-index tramadol use	1.09	1.01	1.18	0.036
Concomitant SNRI use	0.69	0.62	0.76	< .001
Concomitant gabapentin use	0.98	0.86	1.12	0.796
Concomitant opioids use (excluding tramadol)	0.81	0.72	0.90	< .001
Concomitant tramadol use	0.65	0.57	0.75	< .001
Concomitant lidocaine use	0.83	0.68	1.02	0.071
Pre-index number of hospital admissions	1.03	0.99	1.08	0.091
Pre-index number of outpatient visits	1.00	1.00	1.00	0.729
Pre-index number of ER visits	0.85	0.67	1.09	0.205
Pre-index inpatient days	1.00	0.99	1.00	0.004

***NOTE*:** Treatment discontinuation or switch indicates non-persistence. Therefore, dose titration (**bold**) is associated with better persistence.

***Abbreviation*:** PPO, preferred provider organization; HMO, health maintenance organization; POS, point-of-service; CCI, Charlson comorbidity index; SNRI, serotonin and norepinephrine reuptake inhibitors; TCA, tricyclic antidepressants; ER, emergency room.

## Discussion

The goal of clinical management for NeP is to control pain, given that the condition is currently without a curative option. To achieve better pain relief and adherence, the US prescribing information states that pregabalin should be started at 150 mg/day and titrated to 300 mg/day within one week [29]. However, the current study found that most naïve pregabalin users (82%) did not have dose titration within 45 days following the initial dose. Patients who had dose titration tended to have fewer pre-existing conditions, and received alternative treatment before pregabalin, including gabapentin, opioids, and antidepressants. Consistent with the literature on the relationship between dose titration and greater adherence in other disease populations [22, 23], we found that NeP patients who had pregabalin titrated to the target dose were 2.6 times more likely to be adherent, as measured by MPR. Moreover, dose titration was shown to be an independent predictor of greater persistence, indicating that patients who had dose titration were less likely to switch therapy or discontinue pregabalin treatment. Indeed, our study shows that pregabalin users who had dose titration stayed on the treatment for an average of one month longer than patients who did not have dose titration.

One possible explanation for better adherence and persistence among patients with dose titration is that patients who had dose titration are more likely to achieve optimal therapeutic effect. For example, a study using data from 10 clinical trials on pregabalin in multiple indications found that if patients initiated pregabalin treatment at a lower dose and titrated gradually based on the effectiveness, they would be more likely to continue using pregabalin due to better pain relief [37].

An alternative explanation for better adherence and persistence among patients with dose titration is that dose titration can be a proxy of physician-patient relationship. A study of patients with chronic pain found that physician-patient relationship was significantly associated with treatment adherence and outcomes (e.g. pain control, quality of life) [38]. Intuitively, dose titration would require active monitoring of the treatment effectiveness and thus more physician visits and physician-patient communications. Previous research indicated that physician-patient relationship may reflect levels of satisfaction with care, trust in the physician, and patient participation [38]. Future research is warranted to examine how the physician-patient relationship plays a role in dose titration and treatment adherence.

This study has limitations inherent to retrospective claims database analyses that are noteworthy. One of the limitations is that claims databases do not typically contain information on patient-reported pain outcomes. It is possible that some patients might have enough pain relief at a lower dose of pregabalin and therefore did not need dose titration. Future research with information on pain outcomes will be useful to examine the relationship between titration and treatment outcome. In addition, pregabalin dose is recommended to be reduced for patients with chronic kidney diseases [16]. Therefore, it is possible that a subset of the study sample received lower doses due to impaired renal function rather than titration. Future research would benefit if the reason of reduced dose is available in the data source.

## Conclusions

The current study is the first study that examines the effects of dose titration on patient adherence and persistence among patients taking pregabalin for NeP [32]. The positive association between dose titration and treatment adherence that was noted in this study will provide valuable evidence to increase physician awareness of dose recommendations in the prescribing information and to educate patients on the importance of dose titration and adherence and when patients can expect the optimal level of pain relief.
